# groHMM: a computational tool for identifying unannotated and cell type-specific transcription units from global run-on sequencing data

**DOI:** 10.1186/s12859-015-0656-3

**Published:** 2015-07-16

**Authors:** Minho Chae, Charles G. Danko, W. Lee Kraus

**Affiliations:** 1 Laboratory of Signaling and Gene Regulation, Cecil H. and Ida Green Center for Reproductive Biology Sciences, University of Texas Southwestern Medical Center, 75390 Dallas, TX USA; 2 Division of Basic Research, Department of Obstetrics and Gynecology, University of Texas Southwestern Medical Center, 75390 Dallas, TX USA; 3 Baker Institute for Animal Health, College of Veterinary Medicine, Cornell University, 14853 Ithaca, NY USA

**Keywords:** GRO-seq, groHMM, Transcription, Transcription unit, Primary transcript, Gene regulation, Peak calling, Cell type specificity, Enhancer, Primary miRNAs, Long non-coding RNAs (lncRNAs), Enhancer RNAs (eRNAs), ChIP-seq

## Abstract

**Background:**

Global run-on coupled with deep sequencing (GRO-seq) provides extensive information on the location and function of coding and non-coding transcripts, including primary microRNAs (miRNAs), long non-coding RNAs (lncRNAs), and enhancer RNAs (eRNAs), as well as yet undiscovered classes of transcripts. However, few computational tools tailored toward this new type of sequencing data are available, limiting the applicability of GRO-seq data for identifying novel transcription units.

**Results:**

Here, we present groHMM, a computational tool in R, which defines the boundaries of transcription units *de novo* using a two state hidden-Markov model (HMM). A systematic comparison of the performance between groHMM and two existing peak-calling methods tuned to identify broad regions (SICER and HOMER) favorably supports our approach on existing GRO-seq data from MCF-7 breast cancer cells. To demonstrate the broader utility of our approach, we have used groHMM to annotate a diverse array of transcription units (*i.e.*, primary transcripts) from four GRO-seq data sets derived from cells representing a variety of different human tissue types, including non-transformed cells (cardiomyocytes and lung fibroblasts) and transformed cells (LNCaP and MCF-7 cancer cells), as well as non-mammalian cells (from flies and worms). As an example of the utility of groHMM and its application to questions about the transcriptome, we show how groHMM can be used to analyze cell type-specific enhancers as defined by newly annotated enhancer transcripts.

**Conclusions:**

Our results show that groHMM can reveal new insights into cell type-specific transcription by identifying novel transcription units, and serve as a complete and useful tool for evaluating functional genomic elements in cells.

**Electronic supplementary material:**

The online version of this article (doi:10.1186/s12859-015-0656-3) contains supplementary material, which is available to authorized users.

## Background

**Fig. 1:**
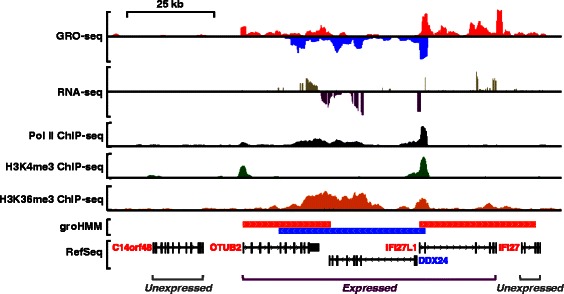
Aligning annotated transcription units with genomic data. Genome browser tracks of various types of genomic data (GRO-seq; polyA+ RNA-seq; Pol II, H3K4me3, and H3K36me3 ChIP-seq) from MCF-7 cells, an estrogen receptor alpha (ERα)-positive human breast cancer cell line. GRO-seq and Pol II ChIP-seq mark the entire transcription unit of actively transcribed genes (“Expressed”). PolyA+ RNA-seq marks exons, 5′ UTRs, and 3′ UTRs of actively transcribed genes. H3K4me3 and H3K36me3 mark the promoters and gene bodies, respectively, of actively transcribed genes. Transcription units called by groHMM using GRO-seq data are shown in comparisons to RefSeq annotations

Recent breakthroughs in high-throughput DNA sequencing technologies have changed our view of the intricate nature of eukaryotic transcriptomes. For example, with adequate sequencing depth, short read RNA-seq can provide a reasonably complete and unbiased identification of protein-coding mRNA isoforms, as well as many types of non-coding RNAs, which can be improved with the use of long read lengths [1–3]. However, RNA-seq enriches for stable mRNAs, which accumulate to steady-state levels in cells. Recent reports indicate that a large number of transcribed regions are rapidly degraded by the exosome [4–6] and are therefore unlikely to be identified by RNA-seq. Therefore, mapping functional elements in cells (*i.e.*, genes, promoters, enhancers, *etc.*) requires the integration of data from large numbers of genomic assays, including ChIP-seq, RNA-seq, DNase-seq, and others [7, 8] (Fig. 1). As a result, complete annotation projects such as ENCODE require a tremendous convergence of resources, vastly exceeding those available to individual labs. Yet large-scale projects such as ENCODE have reported extreme cell type-specificity at many classes of functional elements [7, 9–11], highlighting the need to continue annotating functional elements in new types of cells.

The advent of genomic approaches has allowed detailed characterization of the transcriptomes of mammals and other metazoans. Not surprisingly, such analyses have revealed an enrichment of RNA polymerase II (Pol II) at the promoters and gene bodies of expressed coding and non-coding RNA genes, which correlates with the expression of the cognate RNAs [12–16] (Fig. 1). Unexpectedly, however, recent studies have shown that many other functional elements in the genome, including enhancers, overlap with sites of Pol II loading and active RNA pol II transcription [5, 17–24]. Thus, Pol II marks a surprisingly large number of classes of functional elements across the genome, which together provides an extensive, deep picture of active regulatory and gene expression programs. Mapping sites of active transcription across the genome has been facilitated greatly by a recently developed technology called global run-on sequencing (GRO-seq) [25], which provides a comprehensive ‘map’ of the location and orientation of all three RNA polymerases (Pol I, II, and III) in cells [17, 20, 25].

Given that GRO-seq is a direct measure of transcriptional output, it is well suited for calling active transcription units. However, other approaches, such as RNA-seq and ChIP-seq can be used as well. RNA-seq, which measures steady-state accumulation of RNA, is limited with respect to the calling of transcription units because most mRNAs and lncRNAs are processed to mature forms lacking introns, thus generating sequencing reads that do not cover the entire transcription unit (Fig. 1). ChIP-seq for RNA polymerase II or histone modifications typically associated with the promoters (e.g., H3K4me3) or bodies (e.g., H3K36me3) of actively transcribed genes can also be used for calling transcript units (Fig. 1) [26, 27], but they are surrogates for actual transcriptional output. In contrast, GRO-seq data can robustly identify transcription units (*i.e.*, mRNA and lncRNA genes) [17, 20, 25], as well as the location of distal regulatory elements such as enhancers [17, 18, 21, 24], because it detects the process of transcription before the degradation of unstable RNAs. However, the identification of transcription units from GRO-seq data poses significant new bioinformatic challenges.

Here, we introduce groHMM, a complete pipeline for the (1) accurate identification of the boundaries of transcriptional activity across the genome using GRO-seq data and (2) classification of these transcription units using a database of available annotations, which is provided as an R package in Bioconductor [28]. In addition, we describe novel metrics for the accuracy of transcription unit annotation, which show that groHMM substantially outperforms alternative approaches for identifying both coding and non-coding transcription units. To demonstrate the utility of our approach, we use groHMM to annotate four GRO-seq data sets derived from cells representing a variety of different human tissue types, as well as non-mammalian cells. Our analyses using groHMM, a complete and useful tool for evaluating functional elements in cells, reveal new insights into cell type-specific transcription.

## Implementation

### groHMM, a computational tool for calling transcription units de novo

**Fig. 2:**
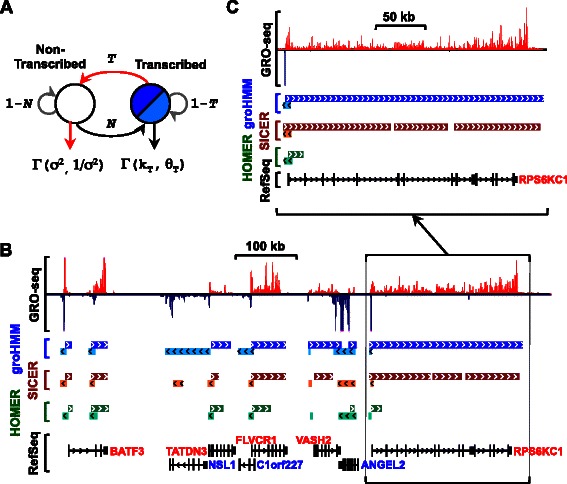
Calling transcription units from GRO-seq data using groHMM. **a** Schematic representation of the groHMM hidden-Markov model approach. The emission probabilities of each state (*i.e.*, transcribed and non-transcribed) were modeled with gamma distributions. Red arrows represent two reserved tuning parameters for the model; *T*, the transition probability of the transcribed state to the non-transcribed state and σ^2^_,_ the variance of the non-transcribed state in a constrained gamma distribution. Γ(σ^2^, 1/σ^2^), constrained gamma distribution of the non-transcribed state; Γ(k_T_, θ_T_), gamma distribution of the transcribed state; *N*, the transition probability of the non-transcribed to the transcribed state. Gray arrows, self-transition probabilities (*i.e.*, transcribed to transcribed or non-transcribed to non-transcribed), which are, by definition, 1-*T* and 1-*N*, respectively. **b** Genome browser tracks of GRO-seq data from MCF-7 cells (*top*) with transcription units called by groHMM, SICER, and HOMER (*middle*), and corresponding RefSeq annotations (*bottom*). **c** Zoomed in view of the browser tracks for the *RPS6KC1* gene from (b)

Transcription units can be identified from various types of genomic data, including GRO-seq, RNA-seq, and ChIP-seq for RNA Pol II, H3K4me3 (promoters), and H3K36me3 (gene bodies) (Fig. 1). We developed an unbiased approach to identify transcription units *de novo* from GRO-seq data using a two-state hidden Markov model (HMM). Our tool, which we call groHMM, is available as an R package in Bioconductor [28]. GroHMM takes as input information about read counts from GRO-seq data in 50 bp windows mapping to the plus and minus strands separately, and then divides the plus and minus strands into states representing “transcribed” and “non-transcribed” regions (Fig. 2a). We used uniquely mapped reads with minimal mismatches allowed as input because multimappers can introduce ambiguity in the HMM (see Methods).

Our HMM is parameterized by probability distributions representing the number of GRO-seq reads each hidden state emits across the genome and by a 2x2 matrix of transition probabilities between the hidden states (Fig. 2a). We used a gamma distribution to model GRO-seq read counts due to its flexibility for representing a variety of probability distributions depending on the values of its parameters, shape (k) and scale (θ). The gamma distribution parameters representing read counts in the transcribed state (k_T_, θ_T_) and the transition probability from the non-transcribed to the transcribed state (*N*) were trained using the Baum-Welch expectation maximization (EM) algorithm. Because GRO-seq has a low background level, we constrained the gamma distribution for the non-transcribed state such that the mean of the distribution became 1 after adding pseudocounts to every window. Self-transition parameters for the transcribed and non-transcribed states are, by definition, 1-*T* and 1-*N*, respectively.

Two parameters of the HMM, representing the transition probability of the transcribed state to the non-transcribed state (T) and the variance of the non-transcribed state (σ2) were held out for tuning using known gene annotations (Fig. 2a). We chose the first tuning parameter, *T*, as a penalty to control the length of the called transcription units. We chose the second tuning parameter, σ^**2**^, to control the variance of the GRO-seq background signal (note that GRO-seq does not have input data, unlike ChIP-seq). Between the two tuning parameters, changing *T* has a larger effect on the length of transcription units than the variance of the constrained gamma distribution (see below). In most of the analyses shown herein, these two tuning parameters were set for mammalian genomes. For non-mammalian genomes with smaller genome sizes and higher gene densities (e.g., *D. melanogaster* and *C. elegans*), a higher range of probabilities for the transition of the transcribed state to the non-transcribed state were more effective (see below). Also, in the groHMM package, users can optimize the tuning parameters by comparing to existing annotations. Details for optimizing the parameters are provided in the tutorial associated with the groHMM tool in Bioconductor [28].

After optimizing the values of the tuning parameters, we used the Viterbi algorithm to obtain a set of called primary transcription units across the genome. Browser track representations of raw strand-specific GRO-seq data from MCF-7 breast cancer cells [17] showing a selected region of the genome, as well as the corresponding transcripts called by groHMM (and two other tools; see below), show that groHMM calls transcripts that align well with RefSeq annotations (Figs 1, 2b, and c).

### Evaluation of transcripts called by groHMM

**Fig. 3:**
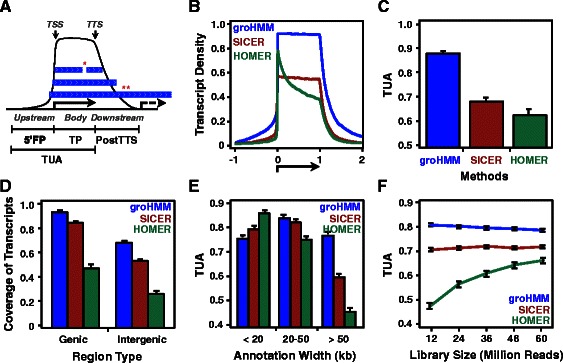
Performance of transcript unit callers. **a** Schematic representation of TUA metrics. We divided non-overlapping gene annotations into three distinct regions: (1) upstream of the transcription start site (TSS), (2) within the gene body, and (3) downstream of the transcription termination site (TTS). We scaled all genes to a uniform length and plotted the frequency with which the called transcription units overlap each position near the gene annotations (see Methods for details). Asterisks represent two possible errors of transcript calling * ‘dissociated annotation error’ and ** ‘merged annotation error.’ The terms are as follows: $\widehat{5\hbox{'}FP}$ (5’ false positive; upstream region), $\hat{TP}$ (true positive; gene body), and $\widehat{PostTTs}$ (downstream of the TTS). **b** Transcript density plot of called transcripts for well-expressed genes (n = 2,060), where expression (*i.e.*, GRO-seq reads) was observed in 100 evenly divided regions (EDR). Transcript density is defined as the number of called transcripts divided by the number of annotations per each genomic location. Ten percent of the transcripts were bootstrapped with replacement (n = 100). **c** TUA metrics for (B), comparing three transcript callers: groHMM, SICER, and HOMER. **d** Coverage of called transcripts compared with actual expression in genic and intergenic regions using a window size of 100 bp for groHMM, SICER, and HOMER. Ten percent of the annotations were bootstrapped with replacement (n = 100). **e** TUAs of called transcripts grouped by annotation widths: short, <20 kb (n = 3,919); medium, 20-50 kb (n = 3,339); and long, >50 kb (n = 4,740) for groHMM, SICER, and HOMER. Annotations with EDR = 10 were used. Ten percent of the transcripts were bootstrapped with replacement (n = 100). **f** TUAs for various sequencing depths for groHMM, SICER, and HOMER. The same optimal values for each method were used. Ten percent of the transcripts were bootstrapped (n = 100) with annotations of EDR = 1

The quality of the boundaries of transcription units called by groHMM from the MCF-7 cell GRO-seq data was evaluated by comparison to known gene annotations. For these analyses, we assumed that the transcripts called by groHMM should largely be in agreement with annotations, when available. Two types of error are naturally defined in this comparison, including cases in which (1) distinct neighboring annotations are merged into a single transcription unit (“merged annotation error”) and (2) a single annotation is broken up into multiple distinct transcription units (“dissociated annotation error”) (Fig. 3a). GroHMM selects the combination of tuning parameters that minimize the sum of these errors after running several settings in an iterative manner.

To compare transcription units called by groHMM with previously annotated genes more rigorously, we devised a measure of mismatch between transcription units and gene annotations where: (1) overlap between the called transcription unit and the annotated gene body, and (2) a lack of overlap between the called transcription unit and the upstream or downstream regions of the annotated gene, provide information on the accuracy of the transcription unit predictions. We divided non-overlapping gene annotations into three distinct regions, all scaled to a uniform gene size: (1) upstream of the transcription start site (TSS), (2) within the gene body, and (3) downstream of the transcription termination site (TTS) (Fig. 3a). For each region, we calculated the proportion (*i.e.*, area) of overlapped transcription units relative to the area of expressed annotations by associating each gene with a best matched groHMM-called transcription unit. We represented these areas as $\widehat{5\hbox{'}FP}$ (5′ false positive), $\widehat{TP}$ (true positive) and $\widehat{PostTTS}$, respectively (Fig. 3a). We assumed that called transcription units overlapping the region upstream of annotated genes are false positives, and that called transcription units overlapping annotated gene bodies are true positives, which provide a measure of sensitivity. The downstream region, however, was not used to define TUA since RNA polymerase II (Pol II) is known to continue transcribing beyond the polyadenlylation site. TUA is a scalar value formally defined as:

$$ \frac{\widehat{TP}+\widehat{5\hbox{'}TN}}{\widehat{TP}+\widehat{FN}+\widehat{5\hbox{'}FP}+\widehat{5\hbox{'}TN}} $$

Where $\widehat{FN}$ (false negative) = 1 - $\widehat{TP}$ for gene bodies. We further restricted TUA to satisfy $\widehat{5\hbox{'}FP}+\widehat{5\hbox{'}TN}$ (5’ true negative) = $\widehat{TP}$, so that the upstream region contributes to TUA only if there is positive number of transcription units in the gene body (*i.e.*, $\widehat{TP}$ > 0). Consensus annotations (*i.e.*, non-redundant annotations from RefSeq and GENCODE), which have non-overlapping genomic coordinates, were used for comparing called transcripts with annotations (see Methods).

### **Results** Performance comparison of groHMM with other transcription unit-calling tools

We selected three additional publically available genomic data analysis tools, SICER, HOMER, and RSEG, and compared the results from these tools to the results from groHMM using the quality metrics described above. All comparisons were done using GRO-seq data from MCF-7 cells [17] (Additional file 1: Table S1). SICER ver. 1.1 [29] was originally designed as a ‘peak caller’ for detecting diffuse enriched regions, such as broad peaks of histone modifications, from ChIP-seq data. It assigns a score for non-overlapping windows, assuming the sequencing reads are distributed under a Poisson model, and combines high scoring windows to form a cluster where gaps are allowed up to given threshold value, which is a user-adjustable free parameter (Table 1). HOMER ver. 4.6 [30] is a method that identifies a sudden increase in GRO-seq signal to denote the start of a transcription unit. The signals are considered artificial spikes if they fail to last over a large distance (Table 1). RSEG ver. 0.4.8 [31] is an HMM-based tool for calling broad peaks of histone modifications from ChIP-seq data (our comparison with RSEG herein are more limited). A fourth tool, Vespucci [32], which defines primary transcription units by assembling reads archived in a relational database, was not evaluated in our comparison. We were unable to get the system working due to technical factors, including its dependence on an external database, PostgreSQL, a commercial cloud environment, and high computational processing times, making it infeasible to test the various free parameters used in the system.

**Table 1:** Parameters of each transcript-calling algorithm tested

Method	Algorithm	Explored parameters	Tested values
groHMM	Hidden-Markov Model	-LtProbB (*T*): Log probability of the transcribed state to non-transcribed state	50..500
		UTS (σ^2^): variance in read counts of the non-transcribed state	5..50
SICER	Clustering approach	windowSize: size of the windows to scan the genome width	200..2000
		gapSize: minimum gap size allowed between windows	1x..10x
HOMER	Transcription model	minBodySize: size of region for transcript body detection	500..5000
		bodyFold: fold enrichment for new transcript detection	2..20

When comparing approaches for calling transcription units from GRO-seq data using tools designed for ChIP-seq data, one must consider the following: First, transcription units are more akin to broad features (e.g., domains of histone modifications, such as H3K36me3; Fig. 1) rather than punctate features (e.g., transcription factor binding sites) that are typically identified in ChIP-seq data. Most ChIP-seq peak callers (e.g., HPeak; [33]) are designed to identify punctate features, although some (e.g., SICER and RSEG; [29, 31]) can identify broad features. Second, most tools designed for use with ChIP-seq data require sequencing data from input samples (*i.e.*, bulk chromatin prior to immunoprecipitation). No such input exists for GRO-seq data due to the nature of the assay [25]. Third, unlike GRO-seq data, ChIP-seq data do not specify the DNA strand (plus or minus). Thus, tools designed for use with ChIP-seq data are not designed to handle genomic data with strand information. Likewise, groHMM is not designed to handle genomic data without strand information. In Additional file 1: Table S2, we provide a list of HMM and non-HMM based broad peak callers and their potential utility for analyzing GRO-seq data.

To compare the performance of each method with groHMM, we ran all methods using the default parameter values (Additional file 1: Table S3). GroHMM returned results with fewer errors and greater transcription unit accuracy. Next, we optimized the tuning parameters of groHMM, SICER, and HOMER independently by exploring 100 parametric models each (see Methods; RSEG has too many parameters to allow efficient fine tuning) (Additional file 1: Figure S1). We determined the overall error, merged annotation error, and dissociated annotation error, as well as called transcript features (e.g., number of transcripts, median transcript length), for groHMM, SICER, and HOMER. Over the range of parameters tested, SICER tended to generate longer and fewer transcripts, while HOMER tended to generate shorter and more numerous transcripts, with groHMM generally falling in between (Additional file 1: Figure S1, A and B). These outcomes are reflected in the errors from each transcription unit caller over the range of parameters tested, with groHMM yielding low overall error rates with the least variance (Additional file 1: Figure S1C), and HOMER yielding the least merged annotation error and the most dissociated annotation errors (Table 2; Additional file 1: Figure S1, D and E).

**Table 2:** Optimal parameter values and error rates for each transcript-calling algorithm tested using GRO-seq data from MCF-7 cells

Method	Parameters	Optimal value	Number of transcripts	Median transcript length (bp)	Error		
					Merged annotation	Dissociated annotation	Rate
groHMM	-LtProbB (*T*)	350	29,639	7,750	1,956	745	0.065
	UTS (σ^2^)	30					
SICER	windowSize	1,200	26,066	13,200	1,602	2,099	0.097
	gapSize	3,600 (3x)					
HOMER	minBodySize	2,500	25,542	4,240	731	1029	0.047
	bodyFold	12					

To evaluate the quality of the called transcription units (*i.e.*, the fidelity of boundaries for gene annotations) for each method, we selected 2,060 highly-expressed mRNAs from the consensus annotations based on a filtering criterion that we call EDR (“Evenly Divided Regions”, defined as the number of equally-sized segments in a gene where at least one GRO-seq read is observed in all the segments. EDR is a measure of robustness and smoothness of gene expression (higher EDR values correspond to more robustly and smoothly expressed genes). Using the optimal parameters for each transcription unit caller (see Methods; Table 2 and Additional file 1: Figure S1) and EDR = 100 for the highly expressed mRNA set, groHMM returned higher TUA values than SICER and HOMER (Figure 3, b and c). Assuming a predicted transcription unit should be in good agreement with a gene annotation only if the gene is transcribed robustly throughout the entire gene body, we expected the TUA values would be positively correlated with the smoothness of expression (*i.e.*, higher EDR values). We found that the TUA values were monotonically increased for groHMM, but not SICER and HOMER, which reached a plateau at EDR = 10 or EDR = 20, respectively (Additional file 1: Figure S2A). Similar results were obtained for a set of highly expressed annotated long non-coding RNAs (lncRNAs) (Additional file 1: Figure S2, B and C). These results suggest that transcription unit boundaries are captured with greater fidelity using groHMM.

Next, we investigated how the called transcription units overlap with expressed consensus annotations by dividing the genome into non-overlapping windows of 100 bp for genic and intergenic regions, and counting the number of windows covered by the called transcripts. The groHMM-called transcription units had better coverage of both genic and intergenic regions than SICER and HOMER (Fig. 3d). We also examined the TUAs with respect to the annotation length (EDR = 10). Whereas HOMER performed slightly better for very short annotations (<20 kb in size), groHMM had a much better performance for both medium (20-50 kb) and long annotations (>50 kb) (Fig. 3e). This was expected because groHMM is tuned using previously annotated genes, which have a median length of 23 kb. There is a more distinctive trade-off between transcript length and performance in SICER and HOMER, while groHMM minimizes this trade-off, robustly covering both short and long transcripts.

Finally, we determined the effect of sequencing depth. We simulated GRO-seq libraries of different sizes by randomly sampling from all chromosomes from the MCF-7 data set up to a total sampled library size of 60 M reads. We then determined the TUA for each transcription unit caller using the optimal values determined above (Fig. 3f). Of the three transcription unit callers, groHMM exhibited the highest sensitivity at the lowest sequencing depths, and the sensitivity was consistent throughout all simulated library sizes. Collectively, our results demonstrate that groHMM is a more versatile, robust, and better-performing tool than SICER and HOMER for calling transcription units.

### Calling transcription units using GRO-seq data from non-mammalian genomes

**Fig. 4:**
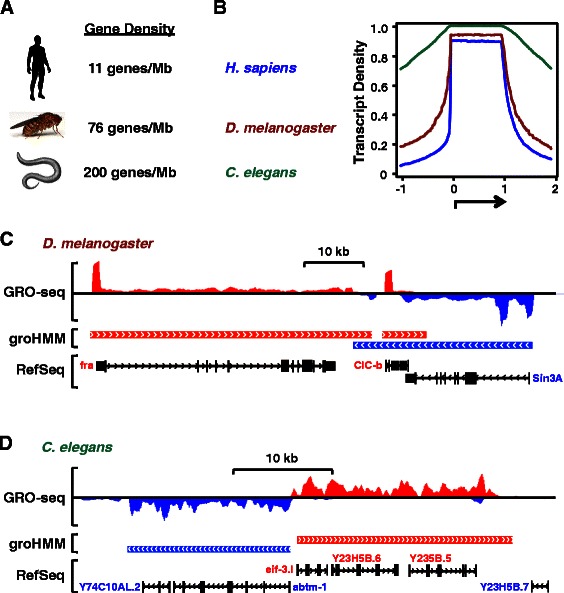
Transcription units called by groHMM using GRO-seq data from *D. melanogaster* and *C. elegans*. **a** Average gene densities for humans (*H. sapiens*), flies (*D. melanogaster*), and worms (*C. elegans*). **b** Transcript density plot for well-expressed transcription units identified by groHMM using optimal parameters. The number of well-expressed genes was 265, 4,524, and 2,060 for *D. melanogaster*, *C. elegans*, and *H. sapiens*, respectively. **c** Sample GRO-seq browser tracks for *D. melanogaster.* Transcription units called by groHMM using GRO-seq data are shown in comparison to RefSeq annotations. **d** Sample GRO-seq browser tracks for *C. elegans*. Transcription units called by groHMM using GRO-seq data are shown in comparison to RefSeq annotations. The gene density is much higher in worms compared to human and flies, resulting in longer groHMM-called transcription units, which merge several gene annotations

GRO-seq is a genomic approach that can be used in non-mammalian organisms, such as flies (*D. melanogaster*) [34] and worms (*C. elegans*) [35]. We used groHMM to call transcription units from publicly available GRO-seq data sets from the fly and worm [35, 36]. As with the analyses using data from human cells, we optimized the parameters (Additional file 1: Tables S4 and S5). Unlike the analysis of data from human cells, we had to explore a larger range of values for the transition probability of the transcribed state to the non-transcribed state (*T*) because of the high gene density in *D. melanogaster* (~76 genes per Mb) and *C. elegans* (~200 genes per Mb) compared to humans (11 genes per Mb) (Fig. 4a). We plotted transcript density as described above for the human data analyses (Fig. 4b). In addition, we determined the number of called transcripts and the error rates (Additional file 1: Tables S4 and S5). Our analyses revealed that groHMM performs well with fly GRO-seq data, but relatively poorly with worm GRO-seq data (Fig. 4, b-d; Additional file 1: Tables S4 and S5). With the fly data, the groHMM-called transcripts matched well with the annotations, while with the worm data, the groHMM-called transcripts typically merged together many annotations (Fig. 4, c and d). The latter is likely due to the high gene density in worms (17-fold greater than humans) (Fig. 4a) and some poorly annotated transcription units for gene clusters, which makes it difficult for groHMM to distinguish distinct genes in gene-dense regions. Overall, we believe groHMM can be useful for the study of some non-mammalian genomes.

### Analyzing and classifying the MCF-7 cell transcriptome

**Fig. 5:**
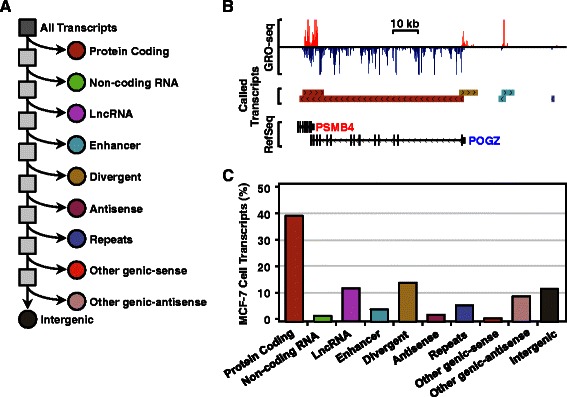
Transcript annotation pipeline and results using GRO-seq data from MCF-7 cells. **a** Schematic representation of a non-overlapping annotation pipeline of called transcripts. **b** Genome browser tracks for various types of transcripts in MCF-7 GRO-seq data. **c** Bar plot showing the fraction of each transcript type among all transcripts called from MCF-7 GRO-seq data using groHMM (n = 31,159). Although plotted in separate bars to match panel (a), all transcripts are uniquely assigned to a single category

Many transcriptomic applications require defining the specific ‘biotype’ of the primary transcripts from predicted transcription units. To illustrate how groHMM can be applied to this task, we classified transcription units called from MCF-7 GRO-seq data [17] (Additional file 1: Table S1) into a set of ten functional classes based on gene annotations, including those producing protein-coding messenger RNAs (mRNAs), non-coding RNAs, lncRNAs, enhancer RNAs, divergent RNAs, antisense RNAs, repeat RNAs, other genic-sense RNAs, other genic-antisense RNAs, and intergenic RNAs, as previously defined [17]. GroHMM predictions were first refined into 31,159 transcription units using a heuristic procedure that reconstructs the boundaries of annotated genes (Additional file 1: Table S1). The non-overlapping annotation pipeline then assigned the cognate transcripts into ten functional classes by comparison to databases of known protein-coding transcripts (including RefSeq GENCODE, and UCSC Genes), lncRNAs (LNCipedia), and transcripts from DNA repeats (RepeatMasker in the UCSC genome browser) (Fig. 5a). Transcripts originating from putative enhancers (*i.e.*, “enhancer transcripts”) were defined as those that are short (<9 kb), bidirectional (*i.e.*, transcribed from both strands of DNA), and partially overlapping, with transcription units whose TSS is located >10 kb from the TSSs or TTSs of annotated genes, as described previously [18]. Although not all bidirectional transcription away from known TSSs indicates an enhancer, since bidirectional transcription that is short in one direction and long in the other may originate from an unnannotated gene [18], short bidirectional intergenic transcription is a good indicator of an active enhancer [18]. This analysis revealed that the most common types of transcription units are those that produce protein-coding RNAs (39 %), divergent RNAs (13 %), intergenic RNAs (13 %), and lncRNAs (12 %) (Fig. 5, b and c).

### Mining public GRO-seq data sets using groHMM

**Fig. 6:**
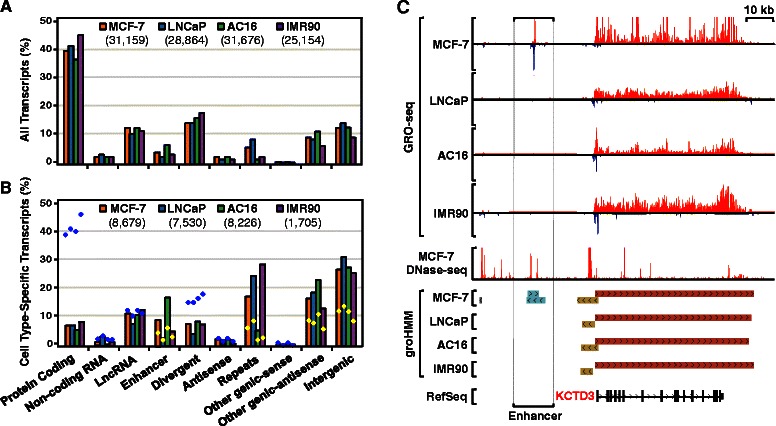
Analysis of cell type-specific transcription using groHMM. **a** Bar plot showing the fraction of transcript types among all transcripts called from GRO-seq data using groHMM in four different cell types (MCF-7, LNCaP, IMR90, AC16). **b** Bar plot showing the fraction of cell type-specific transcripts called from GRO-seq data using groHMM in four different cell types. Blue or yellow dots show the fraction of non-cell type-specific transcripts of each type, which is greater than (*blue dots*) or less than (*yellow dots*) the fraction of cell type-specific transcripts. **c** Genome browser tracks showing examples of a cell type-specific enhancer transcript (MCF-7, marked with brackets) and a non-cell type-specific mRNA transcript (*KCTD3*). DNAse-seq data from MCF-7 cells (ENCODE) is shown for comparison

We systematically compared the transcriptomes of four different cell types, adding three additional publically available GRO-seq data sets to the MCF-7 cell analysis, including data sets from non-transformed cells (cardiomyocytes and lung fibroblasts) and transformed cells (LNCaP prostate cancer cells) (Additional file 1: Table S1). The GRO-seq data from these additional cell lines were processed using groHMM, resulting in distinct sets of transcript for each cell type (Additional file 1: Table S1). The transcriptome profiles were compared across all transcripts or across cell type-specific transcripts (Fig. 6, a and b). Although the total number of transcripts was different for each cell type, the fraction of transcripts within each functional class (e.g., protein-coding, enhancer, intergenic, *etc.*) was similar across the four cell types (Fig. 6a and b). However, the fraction of cell type-specific transcripts in IMR90 cells (~7 %; 1,705 out of 25,154) was significantly lower than in the other three cell types (26 % ~ 28 %; p < 2.2 × 10^-16^, Fisher’s exact test), indicating differences in biology or groHMM sensitivity across cell types. In addition, protein-coding transcripts were significantly depleted from the cell type-specific fraction of the transcriptome in all cell types (p < 2.2 × 10^-16^, Fisher’s exact test), whereas enhancer transcripts were significantly enriched (p < 1.7 × 10^-12^, Fisher’s exact test) (Fig. 6a and b). These observations fit well with the known biology of these transcript types (*i.e.*, enhancers tend to be more cell type-specific than proteins; see the browser tracks in Fig. 6c, for example). As expected, a significant fraction of called enhancer transcripts overlap sites of DNase I hypersensitivity, as determined in analyses of GRO-seq and DNase-seq data from MCF-7 cells (>90 % of the 1,240 enhancer transcripts called; data not shown) (see Fig. 6c, “enhancer”). These results fit well with the known requirement of an open chromatin architecture for enhancer function [19, 37, 38].

### Analysis of cell type-specific enhancers defined by enhancer transcription

**Fig. 7:**
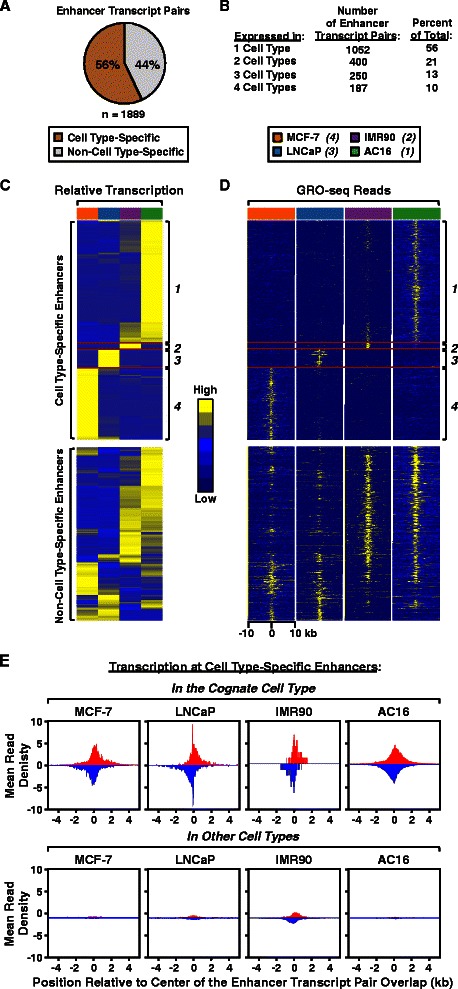
Analysis of cell type-specific enhancers defined by enhancer transcription. **a** Fraction of cell type-specific (n = 1,052) and non-cell type-specific (n = 837) enhancer transcript pairs in the universe of all enhancer transcript pairs called from GRO-seq data using groHMM across four different cell lines (MCF-7, LNCaP, IMR90, AC16). **b** Distribution of enhancer transcript pairs from (A) in one or more cell types. Transcript pairs in one cell type that overlapped a transcript pair in one or more other cell types by at least 20% of their length were counted and summed. **c** Heatmaps showing the relative expression of 1,052 cell type-specific enhancer transcript pairs (*top*) and 837 non-cell type-specific enhancer transcript pairs (*bottom*) after hierarchical clustering analysis. The hierarchical clustering analysis was performed on both the rows and columns using GRO-seq reads on both strands for each enhancer (Ward’s method; [57]). **d** Heatmaps showing normalized GRO-seq read counts for 1,052 cell type-specific enhancer transcript pairs (*top*) and 837 non-cell type-specific enhancer transcript pairs (*bottom*). The order of the enhancers from top to bottom is the same as in (C). Two hundred and fifty bp windows within 10 kb regions from the center of the enhancers are shown. **e** Metagene representations showing the average GRO-seq read distributions ± 4 kb around the center of the enhancer transcript pair overlap for cell type-specific enhancers in their cognate cell type (e.g., MCF-7 cell-specific enhancers in MCF-7 cells) (*top*) or cell type-specific enhancers in other cell types (e.g., LNCaP-, IMR90-, and AC16-specific enhancers in MCF-7 cells) (*bottom*)

Identifying and analyzing cell type-specific enhancers can provide important biological insights. We have previously shown that enhancer transcription detected by GRO-seq is an effective means of identifying and annotating putative enhancers [18]. As noted above, we mined GRO-seq data from MCF-7, LNCaP, IMR90, and AC16 cells using groHMM to identify a universe of 1,889 enhancers (defined by short bi-directional transcription; [18]) across the four cell lines (Fig. 7a). Of these, 56% were cell type-specific (*i.e.*, identified in one cell type only), while the remaining enhancers were detected in two or more cell lines (Fig. 7b). Heat map representations of the relative transcription (Fig. 7c) and the GRO-seq reads within 10 kb of the center of the enhancer transcript pair (Fig. 7d) illustrate clearly the cell type-specificity of many of the enhancers. Metagene plots of the transcription at cell type-specific enhancers in the cognate cell type (*i.e.*, the cell type in which the enhancer is active) versus the other cell types (*i.e.*, those in which the enhancer is inactive) also illustrate the cell type-specificity of many of the enhancers (Fig. 7e).

In order to infer the function of the cell type-specific enhancers that we identified above, we used Gene Set Enrichment Analysis (GSEA) [39]. To do so, we determined the correlation of the transcription of each protein-coding gene with the transcription of each of the 1,052 cell type-specific enhancers. We then ranked the protein-coding genes based on the strength of their correlations and used these rankings to assign enrichment scores for all GSEA categories (*i.e.*, gene ontology, or GO terms) for each enhancer. Next, we performed hierarchical clustering analysis, displaying the normalized GSEA enrichment scores for each enhancer (Additional file 1: Figure S3A; each row is a GO term with its associated normalized GSEA enrichment scores and each column represents an enhancer). This 'guilt-by-association' analysis identified seven clusters (Additional file 1: Figure S3, A and B). A similar analysis of 837 non-cell type-specific enhancers yielded fewer clusters and failed to group the enhancers from each cell type (Additional file 1: Figure S3C). Additionally, the GO terms in the clusters from the cell type-specific analysis in Additional file 1: Figure S3A represent the characteristics of the cell type in which the enhancers are active (Additional file 1: Figure S3, D and E). Finally, we examined how activation of signaling pathways might affect the cell type-specific enhancers. The data from the MCF-7, LNCaP, and AC16 cells gave us a unique opportunity to address this question, given the availability of GRO-seq data sets from hormone-treated cells (MCF-7, estradiol; LNCaP, dihydrotestosterone; AC16, tumor necrosis factor alpha). When compared to the basal (untreated) condition, the treatments affected (either upregulated or downregulated) the transcription of between 25 % and 65 % of the cell type-specific enhancers and putative target genes within a given cell type (Additional file 1: Figure S3, F and G). Collectively, these analyses show that transcripts called by groHMM from GRO-seq data can be used to identify putative cell type-specific enhancers and infer their possible biological functions.

## Discussion

GRO-seq data provides a wealth of information about the cellular transcriptome. Accurately defining various types of transcripts and systematically assigning these transcripts into functional categories poses a great challenge to the research community. In this study, we describe groHMM, a transcription unit identification software package designed for GRO-seq data. In addition, we describe novel metrics for determining the accuracy of transcription unit annotation, which show that groHMM substantially outperforms alternative approaches for identifying both coding and non-coding transcription units. Finally, to demonstrate the utility of our approach, we used groHMM to annotate four GRO-seq data sets derived from cells representing a variety of different human tissue types, focusing on enhancer transcription. Our analyses reveal new insights into cell type-specific transcription.

### Performance of groHMM, a transcription unit identification tool

We used the TUA metric, which compares the overlap of predicted transcription units to protein coding genes, to evaluate the accuracy of groHMM and two alternative transcription unit identification tools. We found that groHMM achieved superior performance compared to both SICER and HOMER when using optimized models for each method. For both mRNA and lncRNA transcription units with lower levels of expression, groHMM was the only method among those tested that showed monotonically increasing performance as a function of the ‘smoothness’ of expression, indicating a high correlation of the groHMM model with gene expression patterns determined from GRO-seq data. A full comparison with HOMER was challenging because of the large number of free parameters that were difficult to explore completely. Therefore, it is possible that parameters which were not fully explored might produce a better outcome. However, adjusting many parameters may not be ideal for typical users since it could easily lead to overfitting. We successfully annotated many transcript types from GRO-seq data sets generated by different labs under a various conditions, including GRO-seq data from non-mammalian cells, further validating the usefulness and generality of groHMM. Together, these results demonstrate that groHMM is the most versatile and accurate of the tools currently available for transcription unit identification using GRO-seq data. There are limitations however; groHMM struggled with the gene dense genome of *C. elegans*, frequently merging separate annotations into a single called transcription unit.

### Annotating and characterizing cell type-specific transcription from GRO-seq data using groHMM

Cell type-specific transcription can be a useful indicator of the biology of different cell types or states, and can even be used to classify cell types into groups with related biology. Our analysis of GRO-seq data across four different human cell lines revealed a considerable amount of cell type-specific transcription for both coding and non-coding transcription units. Cell type-specific transcription was particularly enriched for lncRNA genes, repeat sequences, other genic – antisense sequences, intergenic regions, and enhancers. The cell type-specific transcription of repeat sequences, other genic-antisense sequences, or intergenic regions suggests a further layer of regulatory complexity in the genome that should be explored further. The cell type-specific transcription of enhancers is consistent with observations made in previous studies [5, 11].

A strength of GRO-seq is that activation of gene expression can be measured within the same assay in which one assesses the function of regulatory elements, like enhancers (e.g., by comparing enhancer transcription with protein-coding gene transcription). We used this feature of GRO-seq to assess the functional role of cell type-specific enhancers in the GSEA analysis shown in Additional file 1: Figure S4. Our results suggest that the cell type-specific transcription of regulatory elements and their target genes are tuned to the biology of the particular cell types. Taken together, our analyses of GRO-seq data using groHMM have provided new insights into the function of regulatory elements and the outcomes of cell type-specific gene expression.

### Analysis of cell type-specific enhancers using GRO-seq

Recent studies have placed great emphasis on the identification of cell type-specific enhancers, since they likely control most of the cell type-specific transcription in the cell, including protein-coding and regulatory RNAs (e.g., mRNAs and lncRNAs, respectively). One approach for identifying candidate enhancers is to use DNA sequence features, such as sequence conservation or transcription factor binding motifs [40, 41]. Enhancer prediction based solely on DNA sequence information, however, is challenging since transcription factor binding motifs are short and, thus, occur at many more sites in the genome than are stably bound [42]. Alternatively, features of chromatin, such as chromatin accessibility, histone modifications, or locations of nucleosomes, can be used to identify candidate regulatory sequences [9, 43–45]. All of these genomic approaches for enhancer identification perform better in combination. Other genome-wide approaches use unbiased functional assessment of genomic regions to identify potential enhancer elements [46–48].

We have previously shown that enhancer transcription, as detected by GRO-seq, can be a powerful tool for identifying putative active enhancers [17, 18, 20]. GRO-seq can be a useful tool for detecting the effects of activated cellular signaling pathways (e.g., from hormone treatment) on enhancers [17, 18, 20, 21, 49]. In comparison to other genomic features that have been used to identify “active” enhancers (e.g., transcription factor binding, coregulator recruitment, H3K27ac, DNaseI hypersensitivity, looping to target gene promoters), enhancer transcription may be the most stringent and may give the most robust predictions. In fact, these other enhancers features may be present even when the enhancer has been inactivated and enhancer transcription has been inhibited [18]. Given that enhancer transcription may be the most stringent genomic criterion for calling an active enhancer, it is not surprising that enhancer identification from GRO-seq data yields fewer hits than other genomic approaches. Collectively, our results indicate that analysis of GRO-seq data using groHMM provides a robust approach for identifying putative active enhancers and exploring their functions.

## Conclusions

GRO-seq provides extensive information on the location and function of coding and non-coding transcripts, including primary miRNAs, long non-coding RNAs (lncRNAs), and enhancer RNAs (eRNAs), as well as yet undiscovered classes of transcripts. However, few computational tools tailored toward this new type of sequencing data are available, limiting the applicability of GRO-seq data for identifying novel transcription units. GroHMM, a computational tool in R, which defines the boundaries of transcription units *de novo*, performs favorably when compared to two existing peak-calling methods tuned to identify broad regions (SICER and HOMER). To demonstrate the broader utility of our approach, we have used groHMM to annotate a diverse array of transcription units (*i.e.*, primary transcripts) from four GRO-seq data sets derived from cells representing a variety of different human tissue types, including non-transformed cells (cardiomyocytes and lung fibroblasts) and transformed cells (LNCaP and MCF-7 cancer cells). In particular, we have used groHMM to analyze cell type-specific enhancers as defined by newly annotated enhancer transcripts. Collectively, our results show that groHMM can reveal new insights into cell type-specific transcription by identifying novel transcription units, and serve as a complete and useful tool for evaluating functional genomic elements in cells.

## Methods

### groHMM

The groHMM package was developed in house and is available for download from Bioconductor [28]. The tool was run using both default parameters and tuned parameters as described herein according to the tutorial provided with the groHMM package.

### Mappability

The groHMM tool does not automatically take read mappability into an account. It uses mapped reads from any read mapping tool, with parameters (e.g., number of mismatches) set by the tool. The analyses presented herein manuscript do not take mappability in account for two reasons: (1) the performance gain in transcript calling when considering mappability was minimal (data not shown) and (2) SICER does not consider mappability, so to put the comparisons on equal footing, we did not consider mappability for groHMM and HOMER. To consider mappability in groHMM, one can use existing genome mappability files to mask reads that fall in unmappable regions. The remaining reads can then be used as input to groHMM.

### Data curation and preparation

Publicly available GRO-seq data sets from MCF-7, LNCaP, IMR90, and AC16 cells were downloaded from the NCBI GEO repository using the following accession numbers: MCF-7: GSM678535, GSM678536, GSM678537, GSM678538, GSM678539, GSM678540, GSM678541, GSM678542; LNCaP: GSM686948, GSM686949, GSM686950; AC16: GSM1240738, GSM1240739, GSM1240740, GSM1240741, GSM1240742, GSM1240743, GSM1240744, GSM1240745; and IMR90: GSM340901, GSM340902 (Additional file 1: Table S1). All data except those from the AC16 cells were lifted over to hg19 using the UCSC ‘liftOver’ tool before the analysis. MCF-7 DNaseI-seq data were downloaded using accession number GSM1024784. Non-mammalian GRO-seq data sets were downloaded using the following accession numbers: *D. melanogaster*: GSM1020091, GSM1020092, GSM1020093, GSM1020094; *C. elegans*: GSM1056279, GSM1056282, GSM1056283. The dm3 and ce10 genome assemblies were used for unique mapping of reads in *D. melanogaster* and *C. elegans* data, respectively.

### Additional GRO-seq data analysis tools and tuning parameters

SICER v. 1.1 and HOMER v. 4.6 were downloaded from http://home.gwu.edu/~wpeng/Software.htm and http://homer.salk.edu/homer/download.html, respectively. In order to compare the methods on equal terms, we used two tuning parameters around the default values for each method, thus resulting in one hundred parametric models for each method (Additional file 1: Table 1). The transcription units of each model varied in terms of the number of transcripts detected or the length of the detected transcripts (Additional file 1: Figure S1, A and B). In order to select the optimal model for each transcript caller, we first filtered the models by the median length of the transcripts (within IQR) and subsequently by the number of transcripts (>1.25x and < 1.5x of the consensus annotation). Then, optimal parameters were chosen based on the overall error rate, which is a fraction of the sum of the aforementioned ‘merged annotation error’ and ‘dissociated annotation error’ (Additional file 1: Table 2). For a more precise measure of the dissociated annotation error, we used a well-expressed set of transcripts (n = 11,998) from the consensus annotation, where expression was observed in all 10 evenly divided regions (EDR) of the annotation.

### Consensus annotations

For calculation of the TUA metrics, we used a “consensus” annotation approach where overlapping isoforms of a single annotation are represented by a single genomic interval, using only the interval shared by two or more isoforms. Using shared genomic intervals for isoforms provides representative intervals for each gene, but this approach alone does not resolve all annotation ambiguities because some genes still overlap each other on the same strand (e.g., in the case of redundantly annotated overlapping genomic intervals with different gene symbols). Thus, further overlapping intervals were trimmed at the 3′ end in order to preserve the 5′ transcription start site (TSS) of the interval. The protein-coding genes of RefSeq hg19 (n = 37,560), GENCODE v. 19 Basic (n = 57,584), and UCSC Genes (n = 60,397) were downloaded, combined, and collapsed into a set of 19,834 non-overlapping consensus annotations for all unique gene symbols. These consensus annotations were used for calculating the TUA metrics and for fixing the boundaries of called transcripts with merged annotation errors or dissociated annotation errors. All called transcripts were mapped to annotations one-to-one; if more than one transcript overlapped a single annotation, the best overlapping called transcript was chosen so that dissociated annotation errors were accommodated in the TUA metrics.

### Definitions of transcript categories

Called transcripts were assigned to one of the following ten functional classes, according to the rules defined below, as described previously [17].

(1) Protein coding: A transcript with more than 20 % of its sequence overlapping any well annotated protein-coding gene from RefSeq and GENCODE release 19.

(2) Non-coding transcript: A transcript overlapping an annotated non-coding RNA gene, such as those encoding a miRNA, tRNA, or snRNA, without any restrictions on the size of the transcript or the quality of the overlap using the RNA Genes table from the UCSC genome browser.

(3) lncRNA: A transcript with more than 20 % of its sequence overlapping any well annotated lncRNA from LNCipedia 2.1.

(4) Enhancer: A pair of short (< 10 kb) bidirectionally transcribed intergenic transcripts that do not significantly overlap annotated transcripts.

(5) Divergent: A transcript that overlaps the 5′ promoter driving expression of a primary transcript, such as an mRNA or a lncRNA. Divergent transcripts were only included if (1) >10 % of the transcript overlapped the proximal region of a promoter (±500 bp relative to the TSS) driving expression of a primary transcript >1 kb in size on the opposite strand and (2) the transcript was <50 % of the size of the primary transcript, which effectively excluded divergent enhancer-transcript pairs.

(6) Antisense: A transcript that runs antisense to a protein-coding gene or lncRNA gene and has >20 % of its sequence overlapping >20 % of an annotated protein-coding gene or lncRNA gene on the opposite strand.

(7) Repeat: A transcript with more than 50 % of its sequence overlapping genomic regions identified in the RepeatMasker track in the UCSC Genome Browser.

(9) Other genic – sense/antisense: A transcript that has a poor match to existing annotations. Transcripts in this category overlap any segment of a gene annotation on either strand, but show <20 % matching to the annotation on the same strand (sense) or on the opposite (antisense).

(10) Intergenic: A transcript does not belong to any defined categories above.

### Heatmaps and metagenes

Enhancer transcription heatmaps were generated with Java Treeview, v.1.1.6r4 [50]. Cell type-specific metagenes were created using runMetaGene function in the groHMM package with window size of 100 bp and the sampling option enabled.

### Gene set enrichment and hierarchical clustering analyses

Gene Set Enrichment Analysis (GSEA), v. 2.0.14 [39], was used for the functional study of enhancers using a preranked list generated by excluding terms whose size was >500 or <15 after downloading GO terms for humans from http://download.baderlab.org/EM_Genesets/September_02_2011/Human/symbol/GO/Human_GO_bp_no_GO_iea_symbol.gmt. Normalized enrichment scores (NESs) were used for the subsequent hierarchical clustering analysis after filtering the terms whose NESs were zero for more than 20 enhancers. The heatmap.2 function in the gplots package in R was used for clustering both terms and enhancers using the average linkage option.

### Determining regulation in response to hormone treatment

Regulation in response to hormone treatments was determined using the edgeR package in R [51] with a FDR < 1 % for MCF-7 and AC16 cells. Because of the lack of a biological replicate for the LNCaP cells, housekeeping genes were used to estimate the common dispersion and a p-value < 0.001 was used to call regulation.

## Additional file

Additional file 1:
**The following additional data are available with the online version of this paper.** **(Table S1)**: is a table listing public GRO-seq data sets mined using groHMM in this paper and the outcome of the analyses in various cell types. **(Table S2)**: is a table listing HMM and non-HMM based broad peak callers, and their applicability to the analysis of GRO-seq data. **(Table S3)**: is a table listing the performance of each transcript-calling algorithm tested in detail using GRO-seq data from MCF-7 cells with default parameter values. **(Tables S4 and S5)**: are tables showing a comparison of transcription units called using optimal parameters versus the average of all 50 explored parameter sets for *D. melanogaster* and *C. elegans*, respectively. **(Table S6)**: is a table listing the top ten GO terms for the cell type-specific enhancer clusters. **(Table S7)**: is a table listing the top ten GO terms for the non-cell type-specific enhancer cluster. **(Figure S1)**: is a figure showing the parametric space for explored 100 models comparing three transcript callers: groHMM, SICER, and HOMER. **(Figure S2)**: is a figure showing variations in TUA with gene expression patterns. **(Figure S3)**: is a figure showing a functional analysis of cell type-specific enhancers [52–56].

## Abbreviations

DNaseI: Deoxyribonuclease I
EDR: Evenly divided region
EM: Expectation maximization
$\widehat{FN}$: False negative, gene body
eRNA: Enhancer RNA
GSEA: Gene set enrichment analysis
GO: Gene ontology
GRO-seq: Global run-on coupled with deep sequencing
HMM: Hidden Markov Model
H3K27: Histone H3 lysine 27 acetylation
IQR: Interquartile range
lncRNA: Long non-coding RNAs
mRNA: Messenger RNAs (protein-coding RNA)
NES: Normalized enrichment score
Pol I, II, III: RNA polymerase I, II, III
$\widehat{PostTTS}$: Downstream of the TTS
$\widehat{TP}$: True positive, gene body
TSS: Transcription start site
TTS: Transcription termination site
TUA: Transcription unit accuracy
$\widehat{5\hbox{'}FP}$: 5′ false positive, upstream region
$\widehat{5\hbox{'}TN}$: 5′ true negative, upstream region
