# Supplementary material for: groHMM: a computational tool for identifying unannotated and cell type-specific transcription units from global run-on sequencing data
Source: BMC Bioinformatics. 2015 Jul 16;16:222. doi: 10.1186/s12859-015-0656-3 (PMC4502638; doi:10.1186/s12859-015-0656-3)
Supplement: Additional file 1: — The following additional data are available with the online version of this paper. (Table S1): is a table listing public GRO-seq data sets mined using groHMM in this paper and the outcome of the analyses in various cell types. (Table S2): is a table listing HMM and non-HMM based broad peak callers, and their applicability to the analysis of GRO-seq data. (Table S3): is a table listing the performance of each transcript-calling algorithm tested in detail using GRO-seq data from MCF-7 cells with default parameter values. (Tables S4 and S5): are tables showing a comparison of transcription units called using optimal parameters versus the average of all 50 explored parameter sets for D. melanogaster and C. elegans, respectively. (Table S6): is a table listing the top ten GO terms for the cell type-specific enhancer clusters. (Table S7): is a table listing the top ten GO terms for the non-cell type-specific enhancer cluster. (Figure S1): is a figure showing the parametric space for explored 100 models comparing three transcript callers: groHMM, SICER, and HOMER. (Figure S2): is a figure showing variations in TUA with gene expression patterns. (Figure S3): is a figure showing a functional analysis of cell type-specific enhancers [52–56]. [file 12859_2015_656_MOESM1_ESM.pdf]

Supplemental Data

"groHMM: A Computational Tool for Identifying Unannotated and Cell Type-Specific Transcription Units from Global Run-On Sequencing Data"

Chae *et al.* (2015)

This document contains the following supplemental data: Page

1) Supplemental Tables 2

- Table S1. Public human GRO-seq data sets mined using groHMM 2
- Table S2. List of HMM and non-HMM based broad peak callers and their applicability to the analysis of GRO-seq data 2
- Table S3. Performance of each transcript-calling algorithm tested using GRO-seq data from MCF-7 cells with default parameter values....................3
- Table S4. Comparison of transcription units called by groHMM using optimal parameters versus the average of all 50 explored parameter sets for *D. melanogaster* GRO-seq data 3
- Table S5. Comparison of transcription units called by groHMM using optimal parameters versus the average of all 50 explored parameter sets for *C. elegans* GRO-seq data. 3
- Table S6. Top ten GO terms for the cell type-specific enhancer clusters....................4
- Table S7. Top ten GO terms for the non-cell type-specific enhancer cluster. 6

2) Supplemental Figures 7

- Figure S1. Parametric space explored for 100 models comparing three transcript callers: groHMM, SICER, and HOMER 7
- Figure S2. Variations in TUA with gene expression patterns. 8
- Figure S3. Functional analysis of cell type-specific enhancers in three cell types 9

1) Supplemental Tables

Table S1. Public human GRO-seq data sets mined using groHMM.

Cell Line	Treatments	n	Total Reads	HMM Parameters		Number of Transcripts *(After Correcting for Errors)*
				-LtProbB (*T*)	UTS (σ^2)	
**MCF-7**	E2 for 0,10, 40, 160 min.	2	63,473,424	350	30	31,159
**LNCaP**	DHT for 0, 60 min.	1	17,567,377	150	40	28,864
**AC16**	TNFα for 0, 10, 30, 120 min.	2	108,646,713	250	10	31,676
**IMR90**	N/A	2	10,672,805	150	50	25,154

Table S2. List of HMM and non-HMM based broad peak callers and their applicability to the analysis of GRO-seq data.

Method	HMM-based?	Input Required?	Strand Specific Output?	Usable for GRO-seq data?	Reference
**groHMM**	Yes	No	Yes	Yes	28
**SICER**	No	Optional	No	Yes	29
**HOMER**	No	No	Yes	Yes	30
**RSEG**	Yes	Optional	No	Yes	31
**CCAT**	No	Yes	No	No	53
**ZINBA**	No	Yes	No	No	54
**BroadPeak**	No	Yes	No	No	55
**MACS2** (broad peak run option)	No	Optional	No	No	56

Table S3. Performance of each transcript-calling algorithm tested using GRO-seq data from MCF-7 cells with default parameter values.

Method	Number of Transcripts	Median Transcript Length (bp)	Error			TUA (Transcription Unit Accuracy)
			Merged Annotation	Dissociated Annotation	Total	
**groHMM**	22,686	8,959	1,956	745	2,674	0.896
**SICER**	119,393	2,600	1,602	2,099	9,551	0.533
**HOMER**	129,061	1,573	731	1,029	9,241	0.323
**RSEG**	57,355	4,051	1,617	5,699	5,699	0.801

Table S4. Comparison of transcription units called by groHMM using optimal parameters versus the average of all 50 explored parameter sets for *D. melanogaster* GRO-seq data.

Transcription Units	Number of Transcripts	Median Transcript Length (bp)	Error			Parameters	Value
			Merged Annotation	Dissociated Annotation	Rate		
**Optimal**	15,149	1,650	1,317	601	0.09	-LtProbB (*T*)<br>UTS (σ^2)	50<br>50
**Average of 50 transcript sets**	21,393	1,339	1,231	890	0.10	-LtProbB (*T*)<br>UTS (σ^2)	10..50<br>5..50
**Consensus Annotation**	10,542	2,661	N/A	N/A	N/A	N/A	N/A

Table S5. Comparison of transcription units called by groHMM using optimal parameters versus the average of all 50 explored parameter sets for *C. elegans* GRO-seq data.

Transcription Units	Number of Transcripts	Median Transcript Length (bp)	Error			Parameters	Value
			Merged Annotation	Dissociated Annotation	Rate		
**Optimal**	25,180	1,800	2,989	581	0.10	-LtProbB (*T*)<br>UTS (σ^2)	20<br>50
**Average of 50 transcript sets**	22,009	4,078	2,854	472	0.11	-LtProbB (*T*)<br>UTS (σ^2)	10..50<br>5..50
**Consensus Annotation**	15,297	2,207	N/A	N/A	N/A	N/A	N/A

Table S6. Top ten GO terms for the cell type-specific enhancer clusters.

A. Cluster 1 (n =144)			
**GO Terms**	**ID**	**-log10 p-value**	**Dispensability**
antigen processing and presentation of exogenous peptide antigen	GO:0002478	3	0
proteolysis	GO:0006508	4	0
response to oxidative stress	GO:0006979	4	0
regulation of cell migration	GO:0030334	4	0
response to abiotic stimulus	GO:0009628	4	0.092
response to radiation	GO:0009314	4	0.094
response to biotic stimulus	GO:0009607	3	0.101
vasculature development	GO:0001944	4	0.108
cytokinesis	GO:0000910	3	0.110
cell activation	GO:0001775	4	0.136

B. Cluster 2 (n =86)			
**GO Terms**	**ID**	**-log10 p-value**	**Dispensability**
protein localization to organelle	GO:0033365	4	0
regulation of innate immune response	GO:0045088	4	0
viral reproduction	GO:0016032	4	0.037
cellular macromolecule catabolic process	GO:0044265	4	0.039
chromosome segregation	GO:0007059	4	0.115
interphase	GO:0051325	4	0.121
regulation of ligase activity	GO:0051340	4	0.133
microtubule-based process	GO:0007017	4	0.138
induction of apoptosis	GO:0006917	4	0.147
cytoskeleton organization	GO:0007010	4	0.151

C. Cluster 3 (n =36)			
**GO Terms**	**ID**	**-log10 p-value**	**Dispensability**
RNA splicing	GO:0008380	4	0
cellular component biogenesis	GO:0044085	4	0
establishment of protein localization to organelle	GO:0072594	3	0.031
respiratory electron transport chain	GO:0022904	3	0.117
viral transcription	GO:0019083	3	0.296
RNA catabolic process	GO:0006401	3	0.312
translation	GO:0006412	4	0.341
ncRNA metabolic process	GO:0034660	4	0.347
translational initiation	GO:0006413	4	0.384
ribonucleoprotein complex biogenesis	GO:0022613	4	0.458

D. Cluster 4 (n =57)			
**GO Terms**	**ID**	**-log10 p-value**	**Dispensability**
behavior	GO:0007610	4	0
positive regulation of leukocyte chemotaxis	GO:0002690	4	0.073
C21-steroid hormone biosynthetic process	GO:0006700	4	0.100
synaptic transmission	GO:0007268	4	0.105
cell fate specification	GO:0001708	4	0.113

calcium ion transport	GO:0006816	4	0.249
multicellular organismal signaling	GO:0035637	4	0.391
regulation of exocytosis	GO:0017157	4	0.476
steroid metabolic process	GO:0008202	3	0.506
endocrine process	GO:0050886	3	0.523

E. Cluster 5 (n =112)

GO Terms	ID	-log10 p-value	Dispensability
biological adhesion	GO:0022610	3	0
response to lipid	GO:0033993	4	0
cardiocyte differentiation	GO:0035051	4	0
ribosome biogenesis	GO:0042254	4	0
RNA polyadenylation	GO:0043631	4	0.025
cellular alkene metabolic process	GO:0043449	3	0.056
olefin metabolic process	GO:1900673	3	0.058
positive regulation of calcium ion transport	GO:0051928	4	0.076
sulfur compound metabolic process	GO:0006790	3	0.080
regulation of androgen receptor signaling pathway	GO:0060765	4	0.105

F. Cluster 6 (n =255)

GO Terms	ID	-log10 p-value	Dispensability
circadian rhythm	GO:0007623	3	0
RNA 3'-end processing	GO:0031123	4	0
rhythmic process	GO:0048511	3	0
DNA damage response, signal transduction by p53	GO:0030330	4	0.026
viral reproductive process	GO:0022415	4	0.030
response to ionizing radiation	GO:0010212	4	0.067
bone mineralization	GO:0030282	3	0.078
Golgi vesicle transport	GO:0048193	4	0.100
cell cycle checkpoint	GO:0000075	4	0.108
generation of precursor metabolites and energy	GO:0006091	3	0.108

G. Cluster 7 (n =18)

GO Terms	ID	-log10 p-value	Dispensability
regulation of leukocyte migration	GO:0002685	4	0
extracellular matrix organization	GO:0030198	4	0.101
regulation of behavior	GO:0050795	4	0.130
regulation of angiogenesis	GO:0045765	3	0.138
cellular response to interferon-gamma	GO:0071346	4	0.148
extracellular structure organization	GO:0043062	4	0.374

Table S7. Top ten GO terms for the non-cell type-specific enhancer cluster.

Cluster 1 (n =136)			
**GO Terms**	**ID**	**-log10 p-value**	**Dispensability**
proteolysis	GO:0006508	4	0
microtubule-based process	GO:0007017	4	0
response to abiotic stimulus	GO:0009314	4	0
establishment of protein localization	GO:0048002	4	0
regulation of ligase activity	GO:0016032	4	0.039
response to radiation	GO:0051340	4	0.063
mitotic prometaphase	GO:0000910	3	0.117
chromosome segregation	GO:0007059	4	0.119
induction of apoptosis	GO:0006917	4	0.130
cell division	GO:0006913	4	0.132

1) Supplemental Figures

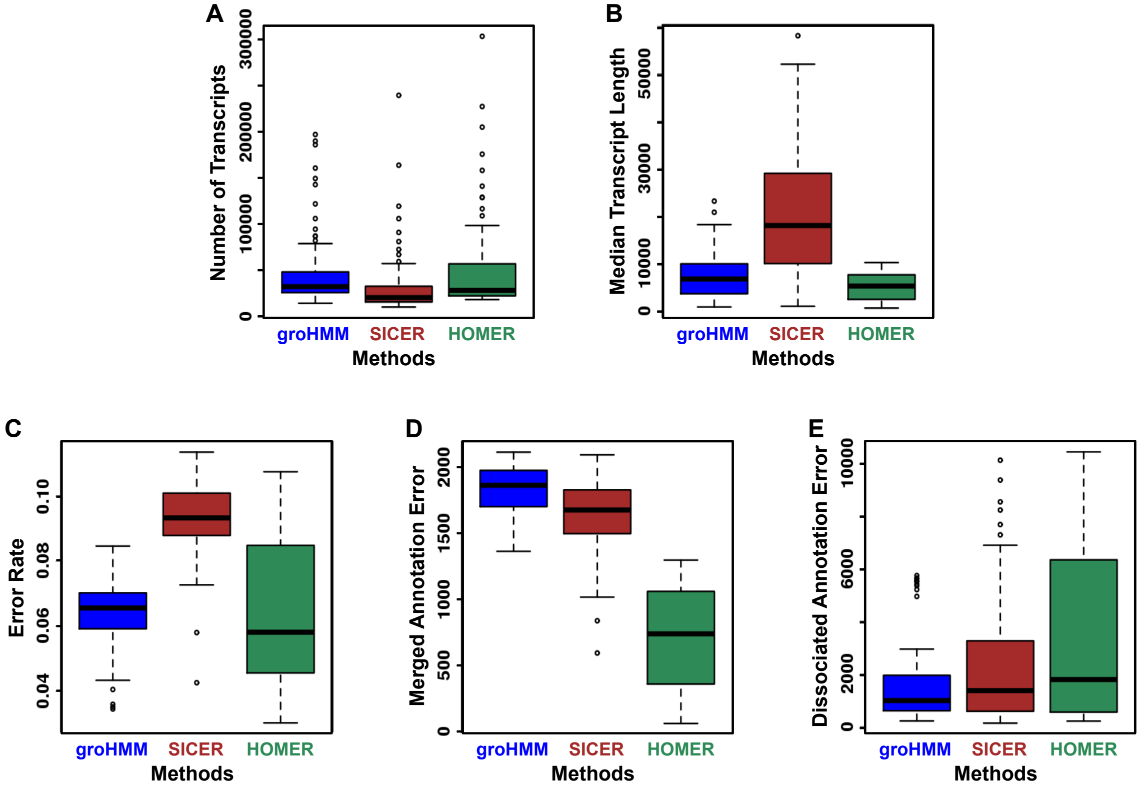

Figure S1. Parametric space for explored 100 models comparing three transcript callers: groHMM, SICER, and HOMER.
(A) Number of transcripts called.
(B) Median transcript length.
(C) Total error rate for two types of error ('merged annotation error' and 'dissociated annotation error').
(D) Number of occurrences of 'merged annotation error.'
(E) Number of occurrences of 'dissociated annotation error.'

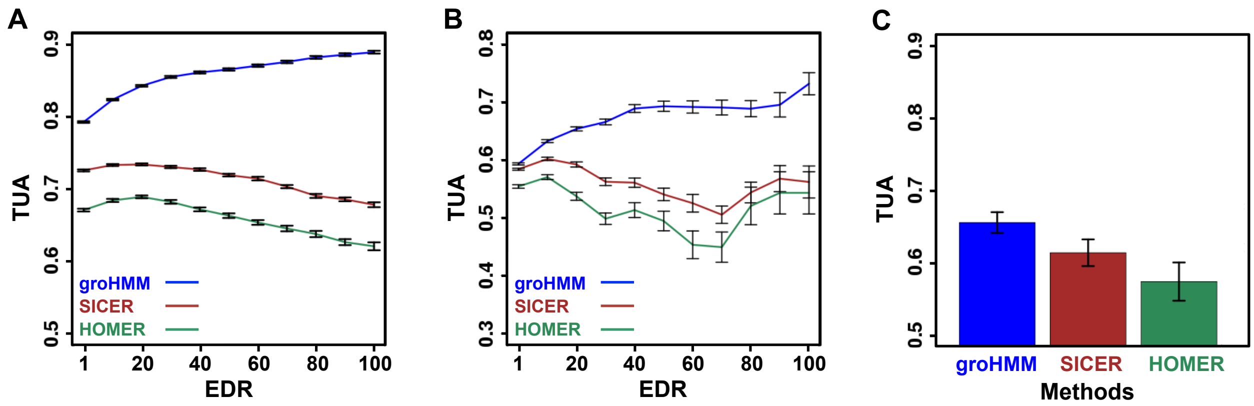

Figure S2. Variations in TUA with gene expression patterns.
(A) Evaluation of TUA when varying EDR (i.e., the smoothness of expression patterns) for mRNA genes.
(B) Evaluation of TUA when varying EDR (i.e., the smoothness of expression patterns) for lncRNA genes.
(C) TUA of called transcripts for well-expressed lncRNA annotations (n = 2,403). Ten percent of the annotations were bootstrapped with replacement (n = 100).

Figure S3. Functional analysis of cell type-specific enhancers in three cell types. *[See following page for the figure]*

In order to infer the function of the cell type-specific enhancers that we identified above, we used Gene Set Enrichment Analysis (GSEA) [39].

(A) Association matrix for the cell type-specific enhancers with functional gene sets. We determined the correlation of the transcription of each protein-coding gene with the transcription of each of the 1,052 cell type-specific enhancers. We then ranked the protein-coding genes based on the strength of their correlations and used these rankings to assign enrichment scores for all GSEA categories (i.e., gene ontology, or GO, terms) for each enhancer. Next, we performed hierarchical clustering analysis, displaying the normalized GSEA enrichment scores for each enhancer. Each row is a GO term with its associated normalized GSEA enrichment scores and each column represents an enhancer. This analysis identified seven clusters (**Table S5**). The GO terms in the clusters represent the characteristics of the cell type in which the enhancers are active. Red = positive association; green = negative association. The clusters were identified by using the cuttree function of R.

(B) Summary heatmap for the clusters shown in (A). The median values of each cluster were used for a more simple visual representation.

(C) Association matrix of non-cell type-specific enhancers with functional gene sets, as in (A). Analysis of 837 non-cell type-specific enhancers yielded fewer clusters and failed to group the enhancers from each cell type (**Table S6**).

(D) The top ten GO terms for cluster 4 (n = 57) summarized by REVIGO. The p-values for all terms were < 0.0001 and ordered by 'dispensability,' which represents the non-redundancy of the term in the group [57]. The cluster 4 enhancers (panel A), which are active in LNCaP cells treated with dihydrotestosterone, are associated with GO terms related to steroid signaling, endocrine processes, and cellular signaling.

(E) Top ten GO terms for cluster 1 (n = 124) in (D) summarized by REVIGO (p-values < 0.001 and ordered by dispensability). The cluster 1 enhancers from the non-cell type-specific enhancer analysis (panel C), which are active in multiple cell types, are associated with GO terms related to a broader array of cellular processes.

(F) Pie charts showing regulation of enhancer transcription by treatment in each cell type (MCF-7, estradiol; LNCaP, dihydrotestosterone; AC16, tumor necrosis factor alpha). Regulation was called using FDR < 1% for MCF-7 and AC16, and p-value < 0.001 for LNCaP with edgeR. The data from the MCF-7, LNCaP, and AC16 cells gave us a unique opportunity to address this questions, given the availability of GRO-seq data sets from hormone-treated cells (MCF-7, estradiol; LNCaP, dihydrotestosterone; AC16, tumor necrosis factor alpha). When compared to the basal (untreated) condition, the treatments affected (either upregulated or downregulated) the transcription of between 25% and 65% of the cell type-specific enhancers within a given cell type, with the effects of the estradiol treatment in MCF-7 cells being most pronounced.

(G) Effects of treatments on mRNA and lncRNA transcript expression in three cell types. Pie charts showing the percent of protein-coding transcripts *(left)* and lncRNA transcripts *(right)* regulated by treatment in each cell type (MCF-7, estradiol; LNCaP, dihydrotestosterone; AC16, tumor necrosis factor alpha). Regulation was called using FDR < 1% for MCF-7 and AC16, and p-value < 0.001 for LNCaP using edgeR. The proportions of regulated protein-coding (mRNA) or lncRNA transcripts were similar to the proportions of regulated enhancer transcripts for each cell types.

Figure S4.

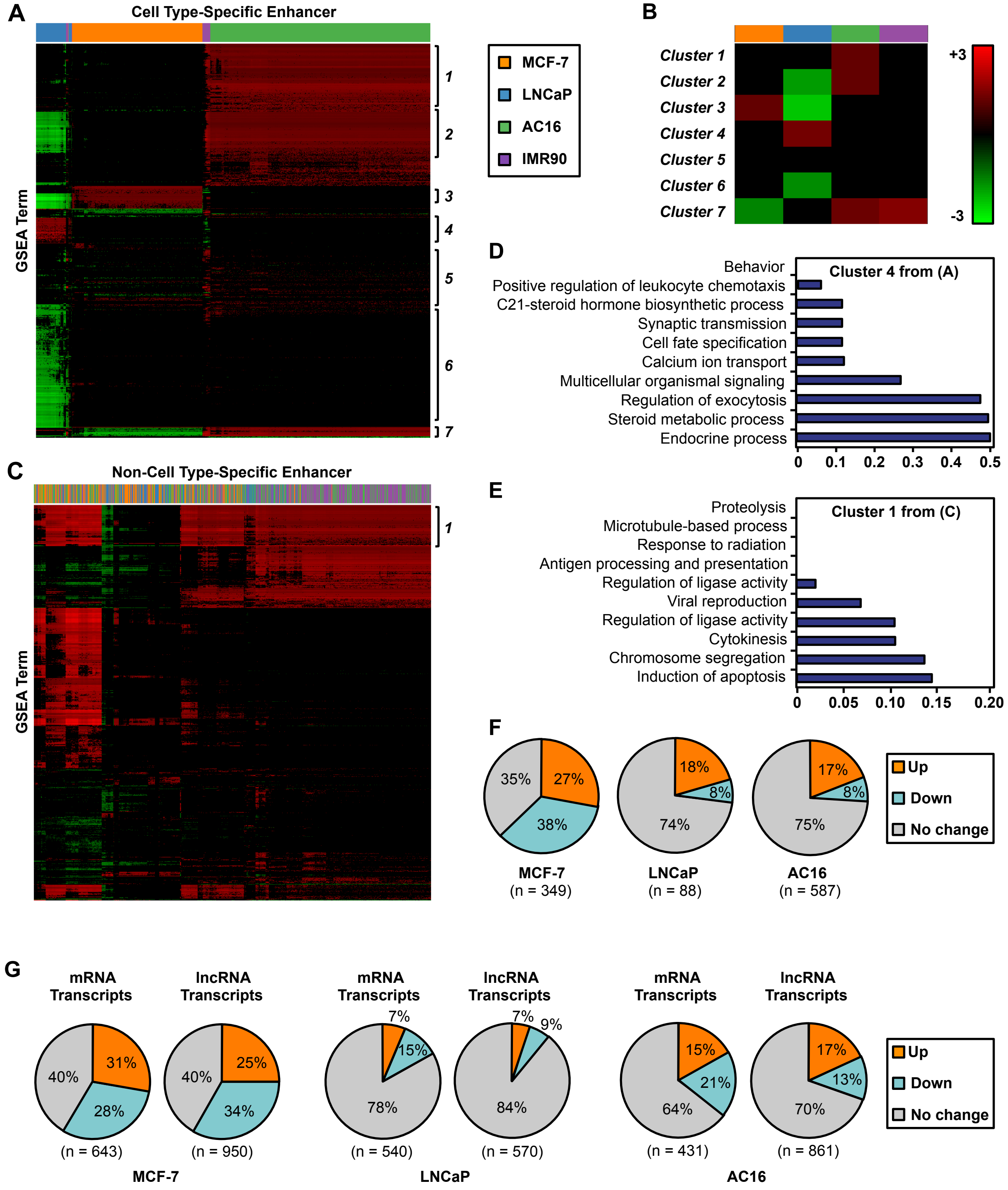
A
Cell Type-Specific Enhancer
GSEA Term
1
2
3
4
5
6
7
MCF-7
LNCaP
AC16
IMR90
B
Cluster 1
Cluster 2
Cluster 3
Cluster 4
Cluster 5
Cluster 6
Cluster 7
+3
-3
C
Non-Cell Type-Specific Enhancer
GSEA Term
1
D
Cluster 4 from (A)
Behavior
Positive regulation of leukocyte chemotaxis
C21-steroid hormone biosynthetic process
Synaptic transmission
Cell fate specification
Calcium ion transport
Multicellular organismal signaling
Regulation of exocytosis
Steroid metabolic process
Endocrine process
0 0.1 0.2 0.3 0.4 0.5
E
Cluster 1 from (C)
Proteolysis
Microtubule-based process
Response to radiation
Antigen processing and presentation
Regulation of ligase activity
Viral reproduction
Regulation of ligase activity
Cytokinesis
Chromosome segregation
Induction of apoptosis
0 0.05 0.10 0.15 0.20
F
35% 27% 38%
MCF-7
(n = 349)
18% 8% 74%
LNCaP
(n = 88)
17% 8% 75%
AC16
(n = 587)
Up
Down
No change
G
mRNA Transcripts
lncRNA Transcripts
mRNA Transcripts
lncRNA Transcripts
mRNA Transcripts
lncRNA Transcripts
40% 31% 28%
(n = 643)
40% 25% 34%
(n = 950)
MCF-7
7% 15% 78%
(n = 540)
7% 9% 84%
(n = 570)
LNCaP
15% 21% 64%
(n = 431)
17% 13% 70%
(n = 861)
AC16
Up
Down
No change
